# Improved machine learning method for analysis of gas phase chemistry of peptides

**DOI:** 10.1186/1471-2105-9-515

**Published:** 2008-12-03

**Authors:** Allison Gehrke, Shaojun Sun, Lukasz Kurgan, Natalie Ahn, Katheryn Resing, Karen Kafadar, Krzysztof Cios

**Affiliations:** 1 Department of Computer Science and Engineering, University of Colorado at Denver, USA; 2 Department of Electrical and Computer Engineering, University of Alberta, Edmonton, Canada; 3 Department of Statistics, Indiana University, Bloomington, IN, USA; 4 Department of Chemistry and Biochemistry, University of Colorado, Boulder, CO, USA; 5 Howard Hughes Medical Institute, University of Colorado, Boulder, CO, USA; 6 Department of Preventive Medicine and Biometrics, School of Medicine, University of Colorado, Denver, CO, USA; 7 Department of Computer Science, Virginia Commonwealth University, Richmond, VA, USA; 8 IITiS, Polish Academy of Sciences, Poland

## Abstract

**Background:**

Accurate peptide identification is important to high-throughput proteomics analyses that use mass spectrometry. Search programs compare fragmentation spectra (MS/MS) of peptides from complex digests with theoretically derived spectra from a database of protein sequences. Improved discrimination is achieved with theoretical spectra that are based on simulating gas phase chemistry of the peptides, but the limited understanding of those processes affects the accuracy of predictions from theoretical spectra.

**Results:**

We employed a robust data mining strategy using new feature annotation functions of MAE software, which revealed under-prediction of the frequency of occurrence in fragmentation of the second peptide bond. We applied methods of exploratory data analysis to pre-process the information in the MS/MS spectra, including data normalization and attribute selection, to reduce the attributes to a smaller, less correlated set for machine learning studies. We then compared our rule building machine learning program, DataSqueezer, with commonly used association rules and decision tree algorithms. All used machine learning algorithms produced similar results that were consistent with expected properties for a second gas phase mechanism at the second peptide bond.

**Conclusion:**

The results provide compelling evidence that we have identified underlying chemical properties in the data that suggest the existence of an additional gas phase mechanism for the second peptide bond. Thus, the methods described in this study provide a valuable approach for analyses of this kind in the future.

## Background

A significant limitation in automated protein identification for high-throughput proteomics research is low discrimination between correct and incorrect peptide assignments obtained by database searches. Recent studies show that prediction of MS/MS fragmentation intensities using the gas phase chemistry simulator in the MassAnalyzer software can achieve accurate results in database searches [1-3]. This simulation is based on kinetic methods, the known gas phase chemical mechanisms for peptide fragmentation [4], and the mobile proton hypothesis [5]. We have shown that a comparison of the observed MS/MS spectra with these theoretical spectra improves peptide identification in the analysis of complex samples [6].

As part of our overall goal to improve the simulator, we developed software (MAE) to evaluate individual fragment ions, and found specific cleavages where the software, based on the kinetic model, did not perform well [6]. In this paper, we focus on one such cleavage, namely that at the second peptide bond from the N-terminus, denoted here as the N2 bond. The simulator in the MassAnalyzer models only one cleavage mechanism for all peptide bonds. The dominant mechanism for peptide bond cleavage yields an oxazolonium product, where the carbonyl oxygen of the preceding peptide bond (in this case, the N1 bond) attacks the back of the carbonyl carbon of the peptide bond [4,5]. Thus, the model is dominated by the parameters driving the large number of cleavages of this type across the whole peptide. However, we observed that the theoretical model under-predicts the intensities at the N2 cleavage site, suggesting that an additional mechanism may be operating. Alternative chemistry at the N2 bond has been proposed, where the peptide amino terminus provides the attacking group to form a diketopiperazine product [7,8].

## Methods

### Data Preparation

Our data is derived from a large shotgun proteomics dataset of an extract of the erythroleukemia cell line K562 grown in suspension as described in [9], with protein profiling as described in [10]. Briefly, gel filtration fractionated proteins were digested with 3% weight trypsin/weight sample in mg protein. Peptides were analyzed by strong cation exchange followed by reverse phase chromatography, on an LCQ mass spectrometer instrument. An important goal is to minimize false positive peptide identifications. To evaluate the results from machine learning algorithms, we needed a high confidence subset of the data, which we achieved through the following five criteria:

1. We required that each MS/MS yielded the same peptide sequence with two search programs, Sequest and Mascot, and that the overall similarity score against the theoretical spectrum was at least 0.54. Previous results showed that this threshold produced nearly complete separation of incorrect and correct assignments with standard peptide MS/MS, and allowed inclusion of cases that have low scores in Mascot and Sequest [10]. The proportions of over- and under-predicted cleavage products were comparable in both the full *vs*. reduced datasets (not shown). Of the alternative MS/MS that yielded different peptide sequences, we selected the one with the highest SumScore. SumScore is a combination of Sequest's XCorr and Mascot's Mowse scores; XCorr is approximately one-half of SumScore and Mowse is approximately seven times SumScore [10]. Although the original dataset contained replicate MSMS spectra, only the highest scoring exemplar of each peptide charge form was included in this analysis.

2. Peptides with observed molecular weight below 950 Dalton were removed, because search programs are notoriously inaccurate for such small peptides.

3. We removed weak spectra, defined in terms of the standard deviation (SD) of the intensities of the fragment ions. A small SD (<1600) of the peptide ion intensities frequently leads to confusion of the noise ions for N2 ions.

4. To minimize the possibility that the N2 ion was produced by contamination of the MS/MS spectrum from a coeluting peptide, we required that the peptide sequence account for at least 90% of the total ion current of the MS/MS spectrum. We utilized the MS/MS feature recognition functions in the MAE software to annotate the fragment ions by ion types, after removing noise ions and combining the isotopic ion clusters to one ion; cf [6]. Fragment ion features were annotated using heuristic rules developed for manual analysis of MS/MS spectra. The misidentification rate is estimated at 1.4%. These rules (and development of MAE software overall) are described in detail in [6].

5. We considered only doubly charged MS/MS, due to the more straightforward interpretation of the chemistry at the N2 cleavage.

The resulting dataset consisted of 12,214 MS/MS spectra, out of the original 69,512 spectra. Criteria 1 and 5 led to the greatest number of exclusions; criteria 2 and 3 had the least effect.

### Pre-processing of intensity information for further analysis

Two types of fragment ions are generated by cleavage at the N2 bond of doubly charged parent ions (a, b, and y ions described in [4,5]):

1. Neutral Loss (referring to the uncharged a/b ions that represent one of the two products of the N2 cleavage)

• a y_(n-2)_^+2 ^ion from an MH_2_^+2 ^parent, which is observed in the middle m/z range of the MS/MS spectrum, where ion yields are high and show good reproducibility.

2. Positive Ion Loss (referring to the singly charged a/b ions that represent one of the two products of the N2 cleavage)

• Large y_(n-2)_^+1 ^ions from MH_2_^+2 ^parents

• Small b_2_^+1 ^or a_2_^+1 ^ions from MH_2_^+2 ^parents (generated at same time as y_(n-2)_^+1 ^ions)

**Figure 1:**
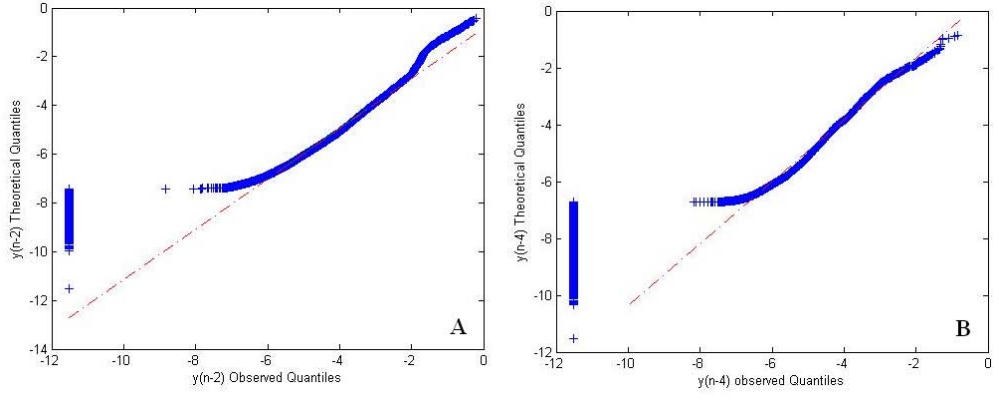
**Q-Q plots**. 1A: QQ plot of the intensities of the y_(n-2) _ions for observed (O) vs. theoretical (T). 1B: QQ plot of the intensities of the y_(n-4) _ions for observed (O) vs. theoretical (T). In each case, the intensities were normalized to the total intensity of the MAE identified ions in the observed spectra or the total intensity of this subset of ions taken from the theoretical spectra (if total intensity of ions in the theoretical is used, the ratios are distorted by the large number of very weak ions that are not usually observed unless a parent ion is very intense). The vertical lines at the left are those cases where the observed is zero and the theoretical is not. The Q-Q plot shows that the N2 and N4 theoretical distributions deviation similarly from the observed distributions in their lower tails, while the upper N2 tail departs from the expected in a way that N4 does not.

This study focuses on the neutral loss cases, because the products from the positive ion loss often are not observed, due to instrument mass biases for both large and small fragment ions. In addition, during manual analyses of data, we often noted large differences between the predicted and observed intensities of the doubly charged cleavage products of the second peptide bond. To confirm this observation in the full dataset, we tested if these differences were observed in other peptide bonds, by comparing the N2 cleavage with the N4 and N5. In each case, intensities of dehydrated/deammoniated forms and their unmodified precursor were combined to produce a single metric for chemical activity of each site (referred to as y_(n-2)_^+2^, y_(n-4)_^+2^, and y_(n-5)_^+2^. A Quantile-Quantile (Q-Q) plot was used to look at the predicted *vs. *observed ratios between the simulated and observed fragment ions generated by cleavage at these sites. This graphical method to compare distributions should be approximately linear if the distributions (predicted ratios and observed ratios) are the same. The theoretical intensities predicted by MassAnalyzer for the y_(n-2)_^+2 ^and y_(n-4)_^+2 ^ions *vs. *the observed ion intensities are shown in Figures 1A and 1B, respectively.

### Evaluating cases where intensity is zero

The Q-Q plot showed the need for proper normalization when the observed and/or theoretical intensities are close to or equal to zero. Such cases may arise if the overall intensity of the MS/MS spectrum is low. Variation in the intensity of the fragmented peptide ion results in observing a different number of the theoretically possible ions. Ions that are relatively low in intensity will be observed in MS/MS of the more intense parent ions, but will be hidden in the noise or may not be detected when the parent ion is lower in intensity. On the other hand, all these less likely products are generated by the kinetic model, where signal to noise ratio is high.

We looked for evidence that the zero observed intensity cases are due primarily to less intense spectra. In Figure 1, where we have programmatically replaced the zero observed value with a very small number, the cases with observed N2 = 0 have log theoretical values ranging from -5 to -15, indicating that these are among the less intense fragment ions in the spectra. Furthermore, when we exclude more spectra with overall low intensity, the number of these cases decreases. These properties are consistent with a detection problem, rather than with a variation in the gas phase chemistry. Therefore, we did not include them in the final dataset, which excluded only an additional 1,387 cases.

We kept those spectra for which both the observed and theoretical intensities are zero (500 examples), because the two are in clear agreement and should be categorized as well-predicted. Furthermore, the size of this subset did not change significantly when we excluded weaker spectra, and the parent ion intensity did not appear as an important classifier in the well-predicted classes. These cases were assigned an Observed/Theoretical (O/T) ratio of 1.0, programmatically.

We classified each fragment ion to indicate whether the ion intensity was well-, over-, or under-predicted (WP, OP, and UP, respectively), based on the ratio between observed and theoretical values. We then built two data models: under-predicted *vs. *well-predicted, and over-predicted *vs. *well-predicted. The "zero issue" predominantly affects the over-predicted vs. well-predicted model. Changing the threshold between over-predicted and well-predicted causes a significant shift in the number of cases classified as over- or well-predicted, whereas changing the under-predicted threshold only causes minor variations in the distribution of the under- and well-predicted classes. In addition, there were many more cases that were under-predicted, making it more difficult to discern clear patterns in the over-predicted vs. well-predicted model, than it is in the under-predicted vs. well-predicted model. The fact that our most significant results are with the under-predicted class is consistent with the need for an additional mechanism in the simulation for this site. For this reason, only results from the well-predicted vs. under-predicted model are described in detail in this paper.

### Attribute Selection

We started with 38 attributes that delineate sequence determinants for the attributes of MS/MS spectra and the amino acid sequences of peptides, including all attributes defined in [11] and additional attributes derived from the observed MS/MS spectra by MAE, as described in [6]. This set includes attributes both from the sequence itself and from the feature recognition function in MAE, as well as other features from the MS/MS spectrum. The 38 attributes are listed in Table 1.

**Table 1:** Attributes used in machine learning algorithms.

**Set A: 27 Attributes derived from sequence**	
AacidN1, AacidN2, AacidN3, AacidN4, AacidN5	First 5 amino acids on N-terminus.
AacidC1, AacidC2	Last 2 amino acids on C-terminus.
HydnN1, HydnN2,..., HydnC2	Hydrophobicity for each of the above seven amino acids.
BasicityN1, BasicityN2,..., BasicityC2	Basicity for each of the above seven amino acids.
Ave_basicity	Average peptide basicity.
NumRs	Number of arginine residues in peptide.
mobileH	Number of basic residues subtracted from peptide charge (indicates existence of mobile proton).
NumHKR_RN2	Number of basic residues to the left of N2 bond.
NumHKR_LN2	Number of basic residues to the right of N2 bond.
OMW	Observed Molecular Weight.

**Set B: 5 Attributes derived from MAE feature recognition function**	

OYMinusB	The balance between y and b ions.
NumIon	Total number of ions.
P_intensity	Intensity of the parent ion.
Osum	(sum of intensity of observed major ions)/(sum of intensity of all ions in the MS/MS output).
Tsum	(sum of intensity of theoretical major ions)/(sum of intensity of all ions)

**Set C: 6 Attributes based on scores generated by database search/sequence validation programs and from our sequence validation methods**	

Mowse	Mascot's score
Xcorr	Sequest's score
SumScore	Summary score; A combination of Sequest's XCorr and Mascot's Mowse score.
PIC	Proportion of the total ion current score for each MS/MS spectrum which accounts for fragment ion assignments.
SIM	Evaluates chemical plausibility based on relative fragment ion intensities when comparing observed MS/MS spectrum to theoretical spectrum.
InterScore	The percentage of observed fragments accounted for by multiple fragmentation events.

As in most data mining applications, we need to identify which attributes of the full set are the key determinants to discriminate between the classes (well- or under-predicted), so we can eliminate irrelevant and/or redundant features. We evaluated correlations among the attribute set and eliminated six redundant attributes including Mowse, XCORR, SIM, Tsum, mobileH, and NumIons. SumScore is a combination of XCORR and Mowse, so the latter two were removed; OMW and the number of ions are highly correlated, so we kept OMW; Osum is correlated with Tsum and SIM, so we kept Osum, because it is an observed measure as opposed to a theoretically derived measure. The number of basic amino acids to the right of the N2 bond is correlated with mobileH. We kept the number of basic amino acids to the right of the N2 bond, because the final MS/MS dataset contained only doubly charged cases, which reduces the range of the mobileH feature.

Attribute selection methods are used to reduce the dimensionality of the data and to simplify the subsequent task of model-building. Determining which selection methods are best for a given data mining application is typically approached experimentally, because different selection algorithms yield varying results, and the results can vary dramatically with small changes in the dataset. We tested for consensus among several attribute selection algorithms using the open-source Weka library [12]. For the under- *vs. *well-predicted subset, three attributes were never selected by any of the algorithms and thus could be eliminated as irrelevant (P_intensity, HydnC2, and BasicityC1). An additional four attributes were eliminated because they were classified as highly unlikely to provide any discriminatory power, due to the fact that they were selected by only one or two of the algorithms (PIC, SumScore, HydnC1, and BasicityN5). Twenty-five attributes remained.

We determined experimentally that the seven attributes which included the first five amino acids on the N-terminus and the last two amino acids on the C-terminus could be removed without loss of accuracy (the total number of correctly classified instances over the total number of instances) in the under- *vs. *well-predicted data. Furthermore, because these attributes represented nominal data, they could not be used in algorithms that require numeric data. The information was also well-represented by the basicity and hydrophobicity of the amino acid in each of the seven positions. We used the remaining 18 attributes for model building. Five features with continuous values (OMW, OYMinusB, interScore, Osum, and Ave_basicity), were discretized using our Class-Attribute Interdependence Maximization (CAIM) algorithm [13,14]. The CAIM, unlike most other discretization algorithms does not require the user to specify a priori the number of discrete intervals; instead it uses class information to calculate their number.

We first performed exploratory data analysis, then for model-building we used supervised methods (a decision tree algorithm, C4.5, and an association rule generating algorithm, as implemented in Weka), plus our own rule generating program, DataSqueezer [14,15].

## Results and Discussion

### Exploratory Data Analysis

**Figure 2:**
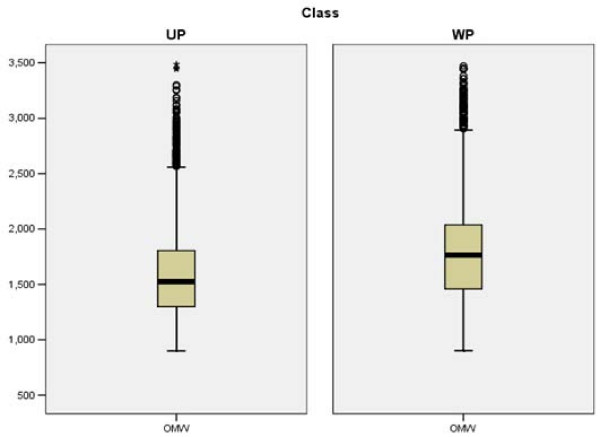
**Boxplots**. Boxplots of observed molecular weights (OMW) for under-predicted ions (UP, left) and well-predicted ions (WP, right). On average, the under-predicted ions have lower OMWs than well-predicted ions.

**Figure 3:**
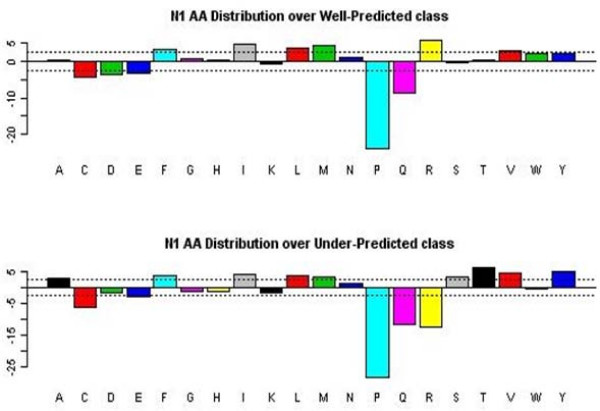
**Amino acid distribution for first position of the observed peptides**. The amino acids are represented by the single amino acid code (A, C,..., Y), and the magnitude of the bar is sqrt(4*observed+2)-sqrt(4*expected+1), where "observed" refers to the observed numbers of the amino acids at the first amino acid position, and "expected" refers to the expected numbers based on the frequency of the amino acids in the data base. Bars that extend beyond the dashed-line limits indicate greater departures from the expected count than would occur simply by chance alone. For well-predicted ions, arginine (R) is more common than in the general data base; for under-predicted ions, it is less common. The given statistic is based on a transformation of an assumed Poisson count that tends to have a standard normal distribution; hence, departures that exceed three in magnitude (location of dashed lines) might be considered unexpected.

**Figure 4:**
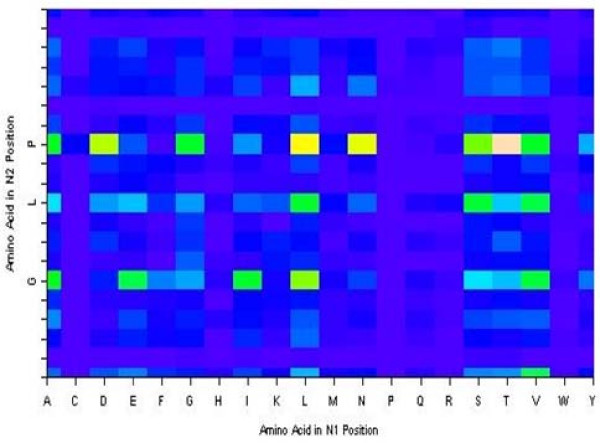
**Observed to theoretical N2 Intensity ratios for all combinations of amino acids in the first and second positions**. The 20 × 20 table of the median values of the N2 ratio of observed intensity to theoretical intensity when the amino acid in the second position is preceded by the amino acid in the first position. The table is displayed as a grid of colored rectangles with the colors corresponding to the values in the table. Glycine, leucine, and proline in the second position consistently have higher observed:theoretical ratios which means the peptides with glycine, leucine, or proline in the second position tend to be under-predicted.

**Figure 5:**
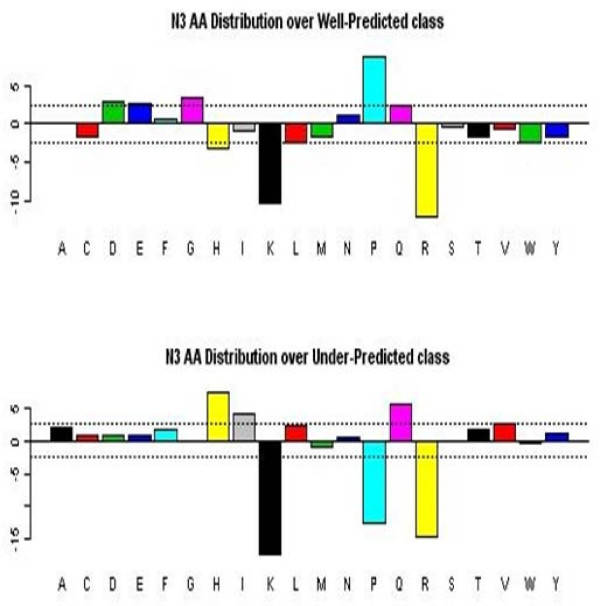
**Amino acid distributions for the third position of the observed peptides**. Frequencies of amino acids in the third position are plotted as described in Figure 3. For well-predicted ions, proline (P) is much more common than in the general data base; for under-predicted ions, it is highly less common. The displayed statistic (see Figure 3 label) is based on a transformation of an assumed Poisson count that tends to have a distribution that is standard normal; hence, departures that exceed three in magnitude (location of dashed lines) might be considered unexpected.

Exploratory data analysis revealed some of the dominant findings used later in model building with decision trees, rule algorithms, and association rules. Figures 2 and 3 demonstrate the tendency of large peptides (OMW values above 1500) and arginine in the N1 position to be well-predicted, respectively. Exploratory analysis also revealed that glycine, leucine, and proline in the N2 position tend to be under-predicted. We generated a 20 × 20 table of amino acids in the N1 position (row) followed by amino acids in the N2 position (column). Each cell in the table held the median value of the observed to theoretical ratio when a given amino acid in N1 is followed by a given amino acid in N2. We frequency-weighted the table to see patterns where the observed to theoretical ratio is unusually high especially compared with the same table charting the amino acids at the N4 bond (a bond that we know is well-modelled by the theoretical prediction). We observed that glycine, leucine, and proline in the N2 position are sequence determinants for under-prediction (Figure 4). The decision tree and association rules also revealed that proline in the N3 position was a sequence determinant for well-prediction (also indicated by analysis in Figure 5).

**Figure 6:**
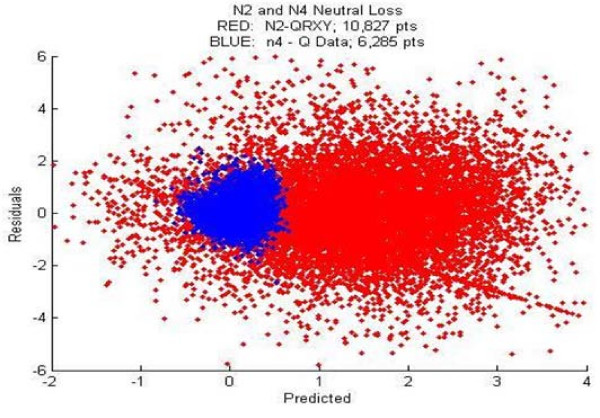
**Linear regression model**. This shows "residuals vs. prediction" in the linear regression model. The blue cluster is the result of regression on data for the N4 bond and the red cluster is the same but with data for the N2 bond. The N2 cluster has a clear spread beyond the N4 cluster center, suggesting the N2 data is not as well predicted as the N4 data. Similar results were observed with the N5 data (not shown).

In addition, we used robust linear regression to fit the logarithm of the ratio of observed intensities to theoretical intensities as a function of the 18 features using the *robustfit *function in Matlab, which uses an iteratively re-weighted least squares algorithm, with the weights calculated at each iteration by applying the *bisquare *function to the residuals from the previous iteration [16]. The results are considerably less sensitive to outliers in the data as compared with ordinary least squares regression. (Recall that 0/0 was set to 1, so its logarithm was set to zero, indicating no difference between them.) We compared the results of robust regression on the N2 dataset against control datasets that contain the same features as the N2 data but with observed and theoretical ions respectively measured and predicted at the N4 bond and at the N5 bond. We chose the N4 and N5 bond for comparison to N2 because we knew experimentally that N4 and N5 did not exhibit the same tendency to under-predict N4 and N5 ion intensities as occurred with those at the N2 site. Figure 6 shows clearly that the distribution of intensities of the N2 ions differs from that of the N4 ions (the N4 and N5 ion intensity distributions are very similar to each other, not shown), indicating that at least one additional chemical mechanism is operating at the N2 bond, that is not currently addressed in the theoretical model.

### Model Building

**Figure 7:**
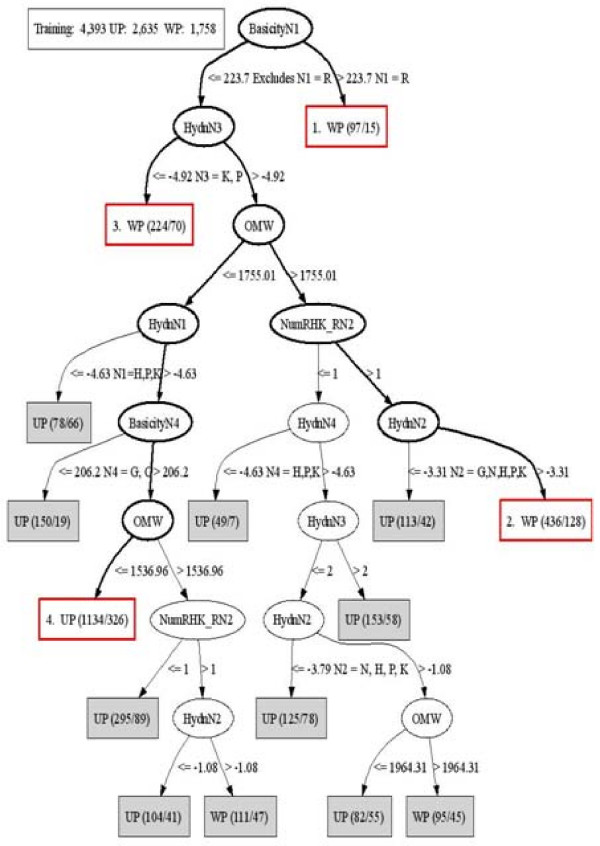
**Decision tree for WP *vs*. UP**. Visualization of Well-predicted (WP) vs. Under-predicted (UP) decision tree, demonstrating the sequence determinants that affect the theoretical prediction at the N2 bond between the second and third amino acid positions. The first number in parenthesis following the classification (either UP or WP) is the number of true positive training points for that rule; that is, the number of instances that are covered by the rule. The subsequent number after the slash indicates the number of counter-instances to the rule; that is, the number of training points that are covered by the rule but are not the class indicated. The four paths through the tree illustrated *via *bolded black edges and non-filled boxes outlined in red are the sequence determinants leading to under- and well-prediction in the theoretical model. The numbers of data points in the tree do not sum to the total shown at the top due to node pruning.

We used different approaches to validate the applicability of supervised and unsupervised techniques to proteomics data and to ensure our results reflected true chemical properties in the data. By and large all the methods were very consistent in their findings. Figure 7 shows a decision tree generated for the under-predicted *vs. *well-predicted data. The dominant theme in the tree is that the decisions are made on the basis of the specific amino acids in the first, second, or third positions in the peptide sequence. This is also represented by the number of basic amino acids to the right of the N2 bond (NumRHK_RN2).

This model suggests several hypotheses:

1. if arginine is the first amino acid, then the N2 cleavage is well-predicted;

2. if:

a. arginine is not the first amino acid;

b. the second amino acid is not glycine, asparagine, histidine, proline, or lysine;

c. the third amino acid is not proline or lysine;

then the N2 cleavage is well-predicteded;

3. if:

a. arginine is not the first amino acid;

b. proline or lysine is in the third position;

then the N2 cleavage is well predicted;

4. if a peptide:

a. has a relatively short sequence;

b. does not have histidine, proline, lysine, or arginine in it's first position;

c. does not have proline or lysine in it's third position;

then it is under-predicted.

We generated many different decision trees using different thresholds on the observed to theoretical N2 ratio to define the well-predicted and under-predicted classes, different attribute subsets, and different parameters of the algorithm. Although the decision trees were always different, some properties were invariant including: (1) two or more basic residues to the right of the N2 bond leads to well-prediction; (2) conversely, zero or one basic amino acids to the right of the N2 bond is an under-prediction indicator; (3) a small OMW leads to under-prediction; and (4) the presence of basic amino acids, proline, or glycine in the second position is an indicator of under-prediction. Peptides with a large OMW tend to be well-predicted, because they are less likely to have the second mechanism in operation (that is, the oxazolonium mechanism predominates in generation of the fragment ions).

These results suggest that the second mechanism might be the generation of a diketopiperazine leaving group (the two amino acid neutrally charged product). This mechanism involves attack of the amino terminal amine on the back of the N2 carbonyl, producing a resonance stabilized six-member ring. The increased frequency of proline in the second position is consistent with the steric effects of proline in that position, which strongly favors the six-membered ring configuration. The diketopiperazine product should also be favoured when the amino terminus is uncharged, or when the N2 bond is less basic [4]; these tendencies can be observed in the decision tree results. In addition, the number of ions (NumIons) is highly correlated with OMW; larger peptides in this dataset are more likely to produce good fragmentation at all peptide bonds, by the oxazolonium mechanism. This would reduce the amount of chemical energy available for the diketopiperazine mechanism.

The DataSqueezer algorithm [15] was used to generate prediction rules for the under-predicted *vs. *well-predicted data. Only strong rules, those that cover at least 5% (UP class) and 3% (WP class) of the corresponding positive data from training data set, were generated. A larger threshold for the under-predicted class was used to accommodate the larger number of samples in this class. Note that the order of attributes/rules does not indicate their importance.

### Machine learning rules for the under-predicted class (true positives 61.9%, false positives 28.8%)

1. If:

a. lysine is the second to last amino acid in the peptide sequence;

b. the number of arginine residues in the peptide sequence is zero;

c. the number of basic amino acids to the left of the N2 bond is zero;

d. number of basic amino acids to the right of the N2 bond is 1;

then it is under-predicted.

2. If:

a. arginine is the second to last amino acid in the peptide sequence;

b. the total number of arginines in the peptide sequence is 1;

c. number of basic amino acids to the right of the N2 bond is 1;

then it is under-predicted.

3. If:

a. OMW is between 900 and 1950;

b. OYMinusB is greater than or equal to -0.10;

c. interScore is between 0 and .16;

d. Osum is between .50 and .98;

e. the number of basic amino acids to the left of the N2 bond is zero;

f. the number of basic amino acids to the right of the N2 bond is 1;

then it is under-predicted.

### Machine learning rules for the well-predicted class (true positives 7.0%, false positives 0.7%)

4. If:

a. proline is the third amino acid in the peptide sequence;

b. arginine is the second to last amino acid in the peptide sequence;

c. the number of basic amino acids to the right of the N2 bond is 1;

then it is well-predicted.

5. If:

a. Arginine is the first amino acid in the peptide sequence;

b. lysine is second to last in the peptide sequence;

then it is well-predicted.

### Comparison of rules generated by the three machine learning approaches

The DataSqueezer rules are consistent with the decision trees and association rules in several ways. In all three methods, determining strong rules for the well-predicted class was more difficult than for the under-predicted class, in part because the under-predicted class had more representation in terms of the number of instances (approximately 65% more cases of under-predicted examples than well-predicted). However, this is an acceptable outcome for the purposes of this study, because we are interested in describing the sequence determinants that lead to under-prediction in order to improve the model in that noticeably deficient area.

Additionally, all three methods demonstrate several general chemical properties: (1) if only one basic residue lies to the right of the N2 bond, the peptide will be under-predicted in the current model; (2) small OMW leads to under-prediction; and (3) arginine in the N1 position and/or proline in the N3 position facilitates a chemical mechanism that is currently well-modelled (i.e., is well-predicted). Although the first rule is difficult to explain, it may represent a higher rate of fragmentation by the oxazolonium mechanism in the bonds to the right of N2, under those conditions. Smaller peptides often show a larger effect of mechanisms other than the oxazolonium mechanism in their MS/MS spectrum, while a strong charged group in the N1 position favours the oxazolonium over the diketopiperazine because the N-terminal amine is more basic. The increased observation of proline in the third amino acid position is generally seen for proline at other positions as well; this is the basis of the so-called proline effect, where fragment ions produced by cleavage at the N-terminal side of a proline are often the most intense ions in an MS/MS spectrum.

The same data were analyzed using association rules (ARs) as implemented in Weka, an unsupervised learning method. ARs are useful for discovering correlations among underlying data. For example, an AR might be stated as: "70% of under-predicted ions also have a basic residue in the N2 position".

The strength of an AR is measured by the support for that rule (i.e., number of cases that support the rule), and by the confidence (i.e., joint occurrence in the relations). ARs require nominal data, which has been categorized, but where the order of the categories is arbitrary.

To compare results between the rule generating programs and the association rule program, we ran association rules as a classifier. In other words, we forced the consequent of the rule (the attribute on the right-hand side of the association) to have only one member: a decision attribute. In all of the following association rules, the class is the consequent of the rule; that is, these are all rules for classifying under- or well-predicted peptides. (Refer to table 1 for translation of attribute names.)

### AR Rules for under-predicted cases

1. If OMW is between 1016.28 and 1550.6 then it is well-predicted.

2. If:

a. OMW is between 1016.28 and 1550.6;

b. the total number of arginines in the peptide sequence is zero or one;

c. number of basic amino acides to the left of the N2 bond is zero;

then it is under-predicted.

3. If OMW is between 1016.28 and 1550.6 and arginine is not the first amino acid in the peptide sequence, then it is under-predicted.

4. If the number of basic amino acids to the left of the N2 bond is zero and the number of basic amino acids to the right of the N2 bond is 1, then it is under-predicted.

5. If:

a. OMW is between 1016.28 and 1550.6;

b. OYMinusB is greater than -0.102481;

c. the number of amino acids to the left of the N2 bond is zero;

then it is under-predicted.

6. If:

a. OMW is between 1016.28 and 1550.6;

b. OYMinusB is greater than -0.102481;

c. arginine is not the first amino acid in the peptide sequence;

then it is under-predicted.

### AR Rules for well-predicted cases

1. If the hydrophobicity of the third amino acid is -4.92, then it is well-predicted.

2. If the basicity of the third amino acid is 214.4, then it is well-predicted.

3. If the number of basic amino acids to the right of the N2 bond is 1, then it is under-predicted.

These rules generated by the unsupervised association rule algorithm are very similar to those implied by the decision trees and the DataSqueezer rule algorithm. Again, it is clear that a small observed molecular weight leads to under-prediction (and conversely, that large observed molecular weight leads to well-prediction). The association rules also detected the importance of arginine is in the first amino acid position.

Additional association rules also support a recurring theme; namely, with only zero or one basic residue (HKR) to the right of the N2 position, the peptide tends to be under-predicted. This finding is related to the mobile proton model [4,5]: the proton required to cleave the peptide bonds is relatively more free to move about the peptide when arginine is present. When present, arginine sequesters the proton to its side-chain, because it has a very high pKa (proton binding constant). If the overall number of protons on the peptide is equal to or less than the number of arginine residues, then protons are available only at local sites. This is most important when the charge on the peptide is equal to or less than the number of arginine residues. In that situation, cleavages are directed mostly by so-called charge-remote mechanisms catalyzed by a proton from the acidic side-chain of aspartate or glutamate. These effects can also occur locally, which probably is the situation in this case, where the N-terminus may be sterically isolated from the rest of the peptide.

The number of basic residues to the left and right of the N2 bond is clearly an important feature of the underlying chemistry. Basic residues to the right of the fragmentation site tend to draw the protons and fragment in well-predicted or chemically well-behaved ways; but basic residues to the left of the N2 bond led to under-prediction. This finding is likely a consequence of our focus on only the neutral-loss cases (where the leaving piece is uncharged), and inadequate modelling of the charge on the N-terminal amine by the Zhang simulation, so that the neutral loss is under-predicted [2]. It may also be due to the difficulty of charging the N-terminus, with the consequence that the uncharged amine can form a stable six-member diketopiperazine with the carbonyl of the N2 bond [4], leading to cleavage of the N2 bond.

Association rules number one and two for well-predicted cases both capture the same relationship (hydrophobicity of proline is -4.92 and basicity of proline is 214.4), that proline in the third amino acid position is associated with well-prediction. This relationship was observed also in exploratory data analysis, the decision tree, and the rule algorithm (see Figures 4 and 6, respectively), and reflects the strong proline effect discussed above. The fact that all three approaches identified, in supervised and unsupervised methods, the strong proline effect gives reassurance that the features uncovered here may in fact be real and not spurious.

We tested the nine features identified by ARs as the most important (OWM, numRs, NumHKR_RN2, numHKR_LN2, BasicityN1, BasicityN3, BasicityN4, HydnN3, and OYMinusB) using the DataSqueezer classifier. With the nine features, DataSqueezer generated 3 rules with 62.7% true positives and 33.3% false positives for the UP class, and 2 rules with 16.7% true positives and 4.5% false positives for the WP class. These results were similar to results obtained with the eighteen attributers, with similar accuracies and true positive rates. This performance is impressive considering that only 9 attributes of the selected 18 were used. In fact, similar results are achieved with just OMW, NumHKR_RN2, NumHKR_LN2, but provides little additional information about the underlying chemistry. The number of basic residues both to the right and left of the N2 bond is a general measure of the amino acid distribution and does not capture the effect of other amino acids like proline, leucine, and glycine.

## Conclusion

The different methods that were used to analyze the data all generated similar results. Importantly, the unsupervised method gave approximately the same results as the supervised methods. This consistency is compelling evidence for the identification of the underlying chemical mechanisms and that the overall analysis, using a combination of chemical knowledge, exploratory data analysis, and machine learning algorithms, is a valuable combination to apply in future studies. Some relationships were indicated more strongly by some methods, confirming that data analysis from different perspectives is useful.

The exploratory data analysis and supervised and unsupervised data mining models elucidated the following pieces of new information/knowledge:

1. Intensities of fragment ions for the N2 cleavage of large OMW peptides are well-predicted.

2. Lysine, proline, histidine, asparagine, glycine, or leucine, in the N2 position leads to under-prediction of the N2 cleavage.

3. Basic amino (HKR) acids in the N1 or N2 position lead to under-prediction. Arginine in the N1 position is a notable exception. This rule is the first split in the decision tree, and as such is a strong sequence determinant. However, this rule is likely influenced by the distribution of the other amino acids in the sequence. In other words, a peptide with arginine as the first amino acid will be well- or under-predicted depending on the distribution of basic residues to the right of the N2 bond as well.

4. The amino acids in the N1, N2, and N3 positions are key determinants.

5. The amino acids in the N5 position or further from the N2 bond do NOT play a role in theoretical prediction at the N2 site.

6. The distribution of basic residues in the sequence to the left and right of the N2 fragmentation site affect the theoretical prediction.

7. Two or more basic amino acids to the right of the N2 site are well predicted; this could be due to the focus on neutral loss of the small product.

The characteristics of MS/MS spectra that cause significant deviations from predicted spectra will be used to improve the kinetic model for theoretical prediction. Further study will be required to confirm our findings derived here from exploratory data analysis and machine learning, and to identify the best combination of analysis tools to reveal new chemical mechanisms. In addition, the analysis methods described in this study provide a reliable work flow for further studies analyzing the other poorly predicted aspects of the MassAnalyzer simulated spectra.

## Abbreviations

Ave_Basicity: attribute representing the average basicity of the peptide; BasicityN1: attribute representing the basicity of the amino acid in the N1 position (have a similar attribute for seven amino acid positions in total); CAIM: Class-attribute Interdependence Maximization; HydnN1: attribute representing the hydrophobicity of the amino acid in the N1 position (have a similar attribute for seven amino acid positions in total); MAE: Manual Analysis Emulator software program; ML: Machine Learning; mobileH: attribute representing to the mobile proton hypothesis; NumIons: attribute representing the number of ions in a peptide; NumRHK_LN2: feature representing the number of basic amino acids (R, H, K) to the left of the N2 bond; NumRHK_RN2: feature representing the number of basic amino acids (R, H, K) to the right of the N2 bond; NumRs: feature representing the number of arginine amino acids in the peptide; OMW: Observed Molecular Weight; OSum: feature representing the observed sum of ion intensities; OYMinusB: feature that represents the balance between y and b ions; PIC: feature representing the proportion of the total ion current score for each MS/MS spectrum which accounts for fragment ion assignments; SIM: Similarity scoring; SumScore: Summary score is a combination of Sequest's XCorr and Mascot's Mowse score; TSum: feature representing the theoretical sum of ion intensities; XCorr: Sequest's cross-correlation score.

## Authors' contributions

AG drafted the manuscript and carried out the data pre-processing and modelling. SS participated in data generation and preparation. LK carried out DataSqueezer analysis. NA participated in the design of the study. KK provided statistical and exploratory data analysis expertise and careful manuscript review. KR and KC conceived of the study and participated in its design and coordination. All authors read and approved the final manuscript.
